# Draft Genome Sequence of *Geobacillus* sp. Isolate T6, a Thermophilic Bacterium Collected from a Thermal Spring in Argentina

**DOI:** 10.1128/genomeA.00743-15

**Published:** 2015-07-16

**Authors:** Elio M. Ortiz, Marcelo F. Berretta, Laura E. Navas, Graciela B. Benintende, Ariel F. Amadio, Rubén O. Zandomeni

**Affiliations:** a Instituto de Microbiología y Zoología Agrícola, Instituto Nacional de Tecnología Agropecuaria (INTA), Buenos Aires, Argentina; b Estación Experimental Agropecuaria Rafaela, Instituto Nacional de Tecnología Agropecuaria (INTA), Santa Fe, Argentina; c Consejo Nacional de Investigaciones Científicas y Técnicas (CONICET), Buenos Aires, Argentina

## Abstract

*Geobacillus* sp. isolate T6 was collected from a thermal spring in Salta, Argentina. The draft genome sequence (3,767,773 bp) of this isolate is represented by one major scaffold of 3,46 Mbp, a second one of 207 kbp, and 20 scaffolds of <13 kbp. The assembled sequences revealed 3,919 protein-coding genes.

## GENOME ANNOUNCEMENT

Some members of the genus *Geobacillus* are thermophilic Gram-positive endospore-forming bacteria, usually isolated from high-temperature environments. Thermophilic strains of this genus have attracted interest as sources of thermostable enzymes with potential biotechnological applications, including proteases, lipases, and glycosyde-hydrolases (1). *Geobacillus* sp. isolate T6 was obtained from a hot water spring in Rosario de la Frontera, Salta, in the northwest of Argentina.

The draft genome sequence was generated using a combined approach between the Roche 454 GS-FLX and Illumina MiSeq platforms (MWG Eurofins), producing unpaired and paired-end reads, respectively. A total of 218,128 single reads with an average length of 350 bp were produced by Roche 454, resulting in 16-fold coverage of the genome. Furthermore, 3,682,898 paired-end plus 3,587,489 singleton reads with an average of 126 bp were obtained using a long jumping distance library with an insert size of 8 kbp from an Illumina MiSeq 2 × 150-bp run, reaching 239-fold coverage. *De novo* hybrid assembly of the reads was performed using Velvet version 1.2.10 (2) with a *k*-mer size of 99. The assembly consists of one major scaffold of 3,468,726 bp (containing 211 contigs), a second scaffold of 207,976 bp, and 20 minor scaffolds of >1 kbp. The order and orientation of the contigs into scaffolds were assessed using Mega BLAST (3) to align the resultant assembly and the genome of *Geobacillus kaustophilus* HTA426, and were visualized using the Artemis comparison tool (4).

The draft genome sequence of *Geobacillus* sp. isolate T6 consists of 3,767,773 bp with an average G+C content of 53%. It was subjected to automated annotation using the RAST server version 2.0 (5) and the NCBI Prokaryotic Genome Annotation Pipeline (PGAP). The tRNA and rRNA genes were predicted by tRNAScan-SE version 1.21 (6) and RNAmmer version 1.2 (7), respectively.

In total, 4,011 genes were identified, including only one copy of 16s rRNA, 13 copies of 23S/5S rRNA, and 78 tRNA genes. RAST predicted 3,919 protein-coding genes, 47% of which were assigned to 452 subsystems. Phylogenetic analysis of the 16S rRNA gene confirmed the affiliation of *Geobacillus* sp. isolate T6 to the genus *Geobacillus*. *Geobacillus kaustophilus* HTA426 (score 531), *Geobacillus thermodenitrificans* NG80-2 (score 495), and *Geobacillus* sp. Y412MC61 (score 495) are the closest neighbor genomes based on RAST analysis.

A group of 878 enzyme-coding genes was appointed as potentially useful in several industries according to their predicted activity, including agriculture, environment, biosensor, biotechnology, medicine, biofuel, food, and other industries. Cloning and expression strategies are under way to confirm the functionality of some of these enzymes. The main goals of our work in gaining genome-based knowledge of thermophilic organisms are to characterize their thermostable enzymes and unravel the metabolic pathways adapted to extreme living conditions.

### Nucleotide sequence accession numbers.

This whole-genome shotgun project has been deposited at DDBJ/EMBL/GenBank under the accession number LDNZ00000000. The version described in this paper is the first version, LDNZ01000000.
